# Manifestações Cardiovasculares Tardias da COVID-19 – Uma Ciência em Construção

**DOI:** 10.36660/abc.20220435

**Published:** 2022-07-29

**Authors:** Sofia Cabral

**Affiliations:** 1 Centro Hospitalar Universitário do Porto Porto Portugal Serviço de Cardiologia, Centro Hospitalar Universitário do Porto, Porto – Portugal; 2 Universidade do Porto Instituto de Ciências Biomédicas Abel Salazar Porto Portugal Instituto de Ciências Biomédicas Abel Salazar, Universidade do Porto, Porto – Portugal

**Keywords:** Covid-19/complicações, Ultrassonografia/métodos, Dilatação Fluxo Mediada, Endotélio Vascular, Inflamação

No início de 2020, o mundo enfrentou o aparecimento de uma nova pandemia, a Doença do Coronavírus 2019 (COVID-19), causada por um novo coronavírus (vírus SARS-CoV-2). Rapidamente descobrimos que, embora o trato respiratório fosse o principal alvo, muitos outros sistemas de orgãos também podiam ser afetados. As manifestações cardiovasculares (CV) da COVID-19 estão entre as mais comuns e temidas e podem se apresentar como lesão miocárdica, arritmias, síndromes coronarianas agudas, insuficiência cardíaca, disfunção vascular e doença tromboembólica.^1^ Tanto a doença cardiovascular preexistente quanto a comórbida bem como a presença concomitante de fatores de risco CV são indicadores de pior evolução e prognóstico da doença.^2^ Digno de nota, o envolvimento CV agudo é um forte preditor independente de mortalidade hospitalar por COVID-19.^3^ A interação entre a infecção e o sistema CV é ainda objeto de estudo mas, além da lesão celular directa pelo vírus, acredita-se que a ativação de vias inflamatórias desempenhe um papel fundamental. Uma descrição detalhada da fisiopatologia do envolvimento CV na COVID-19 está para além do âmbito deste comentário e foi amplamente abordado em outros trabalhos.^4^

Sendo reconhecidas como comuns as desregulação cardíaca e vascular na fase aguda da infecção, as preocupações concentram-se agora nas anormalias CV residuais a longo prazo. Huang et al.,^5^ relataram que mais da metade dos pacientes convalescentes com COVID-19 apresentavam sinais de inflamação miocárdica ou fibrose na ressonância magnética cardíaca (RMC).^5^ Na mesma linha, a meta-análise de Kim et al.,^6^ mostrou que quase metade dos pacientes recuperados de COVID-19 apresentavam um ou mais resultados anormais de RMC.^6^ Da mesma forma, e sem surpresa, a infecção também pode induzir lesão vascular sustentada. À luz do tropismo do SARS-CoV-2 para células que expressam a enzima conversora de angiotensina 2 ligada à membrana, não é de admirar que o endotélio vascular seja um dos principais tecidos envolvidos na infecção. No entanto, os efeitos da infecção por COVID-19 no endotélio vascular e a interação complexa entre os dois ainda são pouco compreendidos. Na sua essência, a homeostase CV depende principalmente do papel do endotélio. Ele regula o tônus vascular, a adesão celular, a tromboresistência, a proliferação de células musculares lisas e, no final, a inflamação da parede do vaso. Sempre que o endotélio é ativado, resultando em disfunção endotelial, a sinalização celular muda de processos vasodilatadores mediados pelo oxido nítrico para processos redox vasoconstritores. Isso pode ocorrer como um fenômeno agudo transitório, com pouca ou nenhuma consequência, ou como uma ativação endotelial prejudicial sustentada, eventualmente levando a um meio pró-aterogênico e pró-trombótico.^7^

A vasomotricidade dependente do endotélio é amplamente reconhecida como um indicador de função endotelial, e a dilatação mediada por fluxo (DMF), originalmente introduzida na década de 1990, é o método não invasivo mais recomendado para avaliá-la.^8^

No estudo de Mansiroglu et al.,^9^ os investigadores usaram a DMF para revelar a presença de envolvimento vascular sutil em pacientes jovens com infecção ligeira por COVID-19 no período inicial pós-infecção. Os autores conduziram uma investigação de caso-controle, de centro único, bem emparelhada, envolvendo 80 pacientes recuperados de Covid-19 em 35 dias (25-178; IIQ: 38,5) após a infecção. Comparados aos controles, o grupo com Covid-19 não apresentou sinais ecocardiográficos de envolvimento cardíaco estrutural ou funcional pela infecção recente. Em contraste, eles exibiram uma resposta de DMF significativamente menor (9,52±5,98 vs. 12,01±6,18, p= 0,010). Estes resultados expandem outros de estudos anteriores ao restringir a inclusão a indivíduos com menos de 45 anos, com infecção ligeira que não justificava a hospitalização. A exclusão de condições concorrentes compartilhando o denominador comum de disfunção endotelial também foi uma característica distinta. Não obstante, dado o curto período de tempo decorrido desde a infecção, é discutível se esses achados podem ser extrapolados como tradutores de qualquer consequência a longo prazo da doença.

Poucos estudos sobre a resposta à DMF analisaram pacientes com COVID-19 jovens e saudáveis, obtendo resultados equivalentes.^10^ Achados semelhantes também foram relatados em um cenário mais amplo. Oikonomou et al.,^11^ estudaram prospectivamente 73 pacientes mais velhos hospitalizados com COVID-19 (37% dos quais necessitaram de tratamento intensivo) e descobriram que a DMF estava significantemente (p < 0,001) diminuída no grupo com COVID-19 (1,65 ± 2,31%) em comparação com uma coorte emparelhada por escore de propensão (6,51 ± 2,91%). Outro aspecto marcante é que essa diferença permaneceu significativa seis meses após a alta (5,24 ± 1,62% e 6,48 ± 3,08%, respectivamente, p=0,01).^11^ Também consistente com estes resultados, o estudo de Gao et al. deu um passo adiante ao demonstrar que a DMF estava reduzida em sobreviventes de COVID-19, mesmo 327 dias após o diagnóstico.^12^

Dada a evidência acumulada, parece inegável a influência a longo prazo na função vascular de uma infecção prévia por COVID-19. No entanto, independentemente dos mecanismos envolvidos, as implicações clínicas dessa afecção vascular são ainda um mistério. Em particular, a susceptibilidade dos sobreviventes de COVID-19 à instalação precoce de aterosclerose é uma preocupação dominante. Da mesma forma, não está claro se esse dano vascular terá impacto em eventos CV futuros. Como resultado, é necessária investigação longitudinal prospectiva adicional de alta qualidade sobre a associação entre disfunção endotelial induzida por COVID-19 e resultados CV a longo prazo. O conhecimento atual relativo às consequências tardias da lesão vascular induzida pela COVID-19 é ainda embrionário. A pesquisa de Mansiroglu et al.^9^ é assim, a par de outras, pioneira neste campo.
