# Supplementary material for: Manifestações Cardiovasculares Tardias da COVID-19 – Uma Ciência em Construção
Source: Arq Bras Cardiol. 2022 Jul 29;119(2):326–7. [Article in Portuguese] doi: 10.36660/abc.20220435 (PMC9363066; doi:10.36660/abc.20220435)
Supplement: Translation

# Translation: COVID-19 and Late Cardiovascular Manifestations – Building Up Evidence

**Authors:** Sofia Cabral

**Affiliations:** 1 Porto Portugal Serviço de Cardiologia, Centro Hospitalar Universitário do Porto, Porto – Portugal; 2 Porto Portugal Instituto de Ciências Biomédicas Abel Salazar, Universidade do Porto, Porto – Portugal

**Keywords:** Covid-19/complications, Ultrasonography/methods, Flow Dilatation, Endothelium, Vascular, Inflammation

In early 2020, the world faced the outbreak of a new pandemic, Coronavirus Disease 2019 (COVID-19), caused by a novel coronavirus (SARS-CoV-2 virus). We soon discovered that while the respiratory tract was the prime target, many other organ systems might also be affected. COVID-19 cardiovascular (CV) manifestations are among the most common and feared and may manifest as myocardial injury, arrhythmias, acute coronary syndromes, heart failure, vascular dysfunction, and thromboembolic disease.^1^ Both pre-existing and comorbid cardiovascular disease and the concurrent presence of CV risk factors are harbingers of a poorer disease course and prognosis.^2^ Notably, acute CV involvement is a strong independent predictor of COVID-19 in-hospital mortality.^3^ The way in which the infection interacts with the CV system remains a subject of study, but apart from direct cell injury by the virus, the activation of inflammatory pathways is thought to play a key role. A comprehensive account of the pathophysiology of CV involvement in COVID-19 is beyond the scope of this commentary and has been extensively covered elsewhere.^4^

While it is now clear that both cardiac and vascular dysregulations are to be expected during the active stage of the infection, concerns now focus on long-term residual CV abnormalities. Huang et al.^5^ reported that more than half of convalescent COVID-19 patients displayed signs of myocardial inflammation or fibrosis on cardiac magnetic resonance (CMR) imaging.^5^ Similarly, the meta-analysis by Kim et al.^6^ showed that nearly half of recovered COVID-19 patients exhibited one or more abnormal CMR results.^6^ Likewise, and somewhat unsurprisingly, the infection may also induce enduring vascular injury. In light of the tropism of SARS-CoV-2 for membrane-bound angiotensin-converting enzyme 2-expressing cells, it is no wonder that the vascular endothelium is a major player in the infection. Nonetheless, the effects of COVID-19 infection on vascular endothelium and the complex interplay between the two are still poorly understood. In essence, CV homeostasis is primarily dependent on the role of the endothelium. It regulates vascular tone, cell adhesion, thromboresistance, smooth muscle cell proliferation, and ultimately, vessel wall inflammation. Whenever the endothelium is activated, resulting in endothelial dysfunction, cell-signaling shifts from nitric oxide - mediated vasodilatory to vasoconstrictor redox processes. This can occur as a transient acute phenomenon, with little to no consequences, or as a sustained detrimental endothelium activation, eventually leading to a pro-atherogenic, pro-thrombotic milieu.^7^

Endothelium-dependent vasomotion is widely acknowledged as a surrogate marker of endothelial function, and flow-mediated dilation (FMD), originally introduced in the 1990s, is the most endorsed non-invasive method for assessing it.^8^

In the study by Mansiroglu et al.^9^ investigators used FMD to uncover subtle vascular involvement in young, mildly affected COVID-19 patients in the early post-infection period. The authors conducted a single-center, well-matched case-control investigation involving 80 recovered Covid-19 patients within 35 days (25-178;IQR: 38.5) of infection. Compared to the controls, the Covid-19 group had no echocardiographic signs of structural or functional cardiac affection from the recent infection. In contrast, they exhibited a significantly lower FMD response (9.52±5.98 *versus* 12.01±6.18, p= 0.010). These results expand on previous studies by restricting enrollment to individuals younger than 45, with mild infection not warranting hospitalization. The exclusion of competing conditions sharing the common denominator of endothelial dysfunction was also a distinctive feature. Notwithstanding, given the short timeframe from infection, it is arguable whether these findings extrapolate to any long-term consequence of the disease. Few studies on FMD response looked at young, otherwise healthy COVID-19 patients, getting equivalent results.^10^ Similar findings have also been reported in a broader setting. Oikonomou et al.^11^ prospectively studied 73 elder COVID-19 hospitalized patients (37% of whom required intensive care treatment) and found that FMD was significantly (p < 0.001) impaired in the COVID-19 group (1.65 ± 2.31%) compared with a propensity score-matched cohort (6.51 ± 2.91%). Another remarkable aspect is that this difference remained significant six months after discharge (5.24 ± 1.62% and 6.48 ± 3.08%, respectively, p=0,01).^11^ Consistent with these results, the study by Gao et al., went a step further by demonstrating that FMD was reduced in survivors of COVID-19, even 327 days after diagnosis.^12^

Given the accumulating evidence, the long-term influence of a prior COVID-19 infection on vascular function is compelling. However, regardless of the involved mechanisms, the clinical implications of this vascular affection remain a mystery. Of special concern is the prospect of early onset of atherosclerosis in survivors of COVID-19. In this regard, it is unclear whether this vascular damage will impact on future CV events. As a result, further high-quality prospective longitudinal research on the association between COVID-19-induced endothelial dysfunction and long-term CV outcomes is necessary. As we stand, we are at the outset of unfolding the full long-term consequences of Covid-19-induced vascular injury. The research by Mansiroglu et al.^9^, along with others’, is ground-breaking.
